# Beyond Green Chemistry: The Emerging Physics of Non-Isocyanate Polyurethanes

**DOI:** 10.3390/ma19132732

**Published:** 2026-06-25

**Authors:** Konstantinos N. Raftopoulos

**Affiliations:** Department of Chemistry and Technology of Polymers, Faculty of Chemical Engineering and Technology, Cracow University of Technology, Warszawska 24, 31-155 Kraków, Poland; konstantinos.raftopoulos@pk.edu.pl

**Keywords:** non-isocyanate polyurethane, polyhydroxyurethane, hydrogen bonding, microphase separation, bond-exchange, vitrimer, glass transition, water sorption

## Abstract

Non-isocyanate polyurethanes (NIPUs) produced by the aminolysis of cyclic carbonates are often presented as safer and more sustainable alternatives to conventional polyurethanes. Their monomer sourcing and synthetic pathways are by now fairly well explored, but the physical principles controlling their properties remain much less understood. This perspective challenges the notion that these materials follow the paradigm of conventional polyurethanes. Emphasis is placed on the hydroxyl group formed next to the urethane moiety, which distinguishes these materials from conventional polyurethanes and makes them more precisely poly(hydroxy urethanes). The available evidence indicates that this pendent hydroxyl is not a minor structural detail but a central actor affecting hydrogen bonding, microphase separation, and through them, many macroscopic physical properties of NIPUs, such as glass transition, mechanical response, water uptake and reprocessability. In addition, it enables thermally activated bond-exchange reactions, which dynamically change chain connectivity and, in networks, topology. As a result, concepts borrowed from conventional segmented polyurethanes cannot be transferred directly to non-isocyanate ones. Instead, a new, physics-oriented predictive framework is the necessary next step for the rational design of non-isocyanate polyurethanes. Such a framework should take bond-exchange reactions into account and connect molecular structure and thermal history with the macroscopic physical properties.

## 1. Introduction: Non-Isocyanate Polyurethanes in Context

When I first heard about non-isocyanate polyurethanes, I was not impressed. As a polymer physicist, the details of polymer synthesis are less important to me than the final structure. So, I treated non-isocyanate polyurethanes as “polyurethane business as usual” and dealt with other things while my chemist colleagues struggled with the—admittedly non-trivial—synthesis. It was only a few months later that I noticed two things almost by chance: an unusual drop in a thermogravimetric curve being recorded in the laboratory, and a hydroxyl adjacent to the urethane group, in a chemical structure drawn by a PhD student. It turned out that this hydroxyl, an apparently small structural difference, gives NIPUs new properties but also raises new challenges in understanding structure–property relationships in such systems. In this text, I would like to address the broader question of how far these non-isocyanate polyurethanes, essentially poly(hydroxy urethanes) (PHUs), can be understood within the paradigm of conventional polyurethanes, and where that paradigm must be extended.

The central argument is that NIPUs obtained by aminolysis of cyclic carbonates should not be viewed simply as conventional polyurethanes prepared by a safer synthetic route. The hydroxyl group formed together with the urethane linkage changes the hydrogen-bonding landscape, the ability of the chains to organize into hard and soft domains, the interaction with water, and the chemistry available during thermal treatment. Therefore, the physical model developed for conventional segmented polyurethanes is an essential starting point, but it is not sufficient.

**Figure 1:**
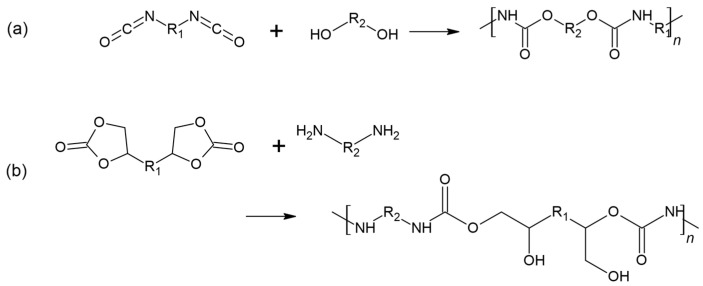
Chemical reactions for the synthesis of (**a**) conventional polyurethanes and (**b**) non-isocyanate polyurethanes/poly(hydroxy urethanes) by aminolysis of cyclic carbonates. In (**b**) note that the resulting hydroxyl may be primary or secondary, depending on the ring-opening pathway.

In order to put everything into perspective, we need to start in the early 1930s, when Carothers and his colleagues in the laboratories of DuPont in Delaware, USA laid the foundations of what is now called step-growth polymerization [1,2,3]. They developed its theoretical framework alongside the development of some of the most iconic polymers, namely polyamides and polyesters. A little later, in 1937, in IG Farben labs in Leverkusen, Germany, Otto Bayer and colleagues followed suit, studying the reaction of diisocyanates with diols, giving rise to an equally iconic class of polymers, namely polyurethanes [4,5] (Figure 1a). Alongside them, the strongly related class of polyureas was developed by the reaction of diisocyanates and diamines.

The inherent beauty of step-growth polymerization is the ability to synthesize polymers with tailored properties by proper selection of prefabricated monomers and macromonomers. Using Bayer’s own words, “*one could prepare macromolecules of any desired configuration using diisocyanates and premanufactured co-reactants, just as an architect can design beautiful buildings*” [4]. Polyurethanes not only became a commercial success, but almost a century later they continue to evolve and adapt to modern needs and concerns [6,7,8]. The scientific and technological relevance of polyurethanes arises from the versatility of available raw materials, but most importantly from their segmental architecture. This architecture leads to a tunable microphase-separated morphology, which has a strong impact on their mechanical, thermal, surface, and functional properties [9,10].

However, polyurethanes and polyureas were faced from the very beginning with a crucial safety concern. Again, in Bayer’s own words, referring to the post-war era when he was looking for strategic alliances, “*even companies who were doing research in the diisocyanate field did not believe in technical production possibilities for the diisocyanates. They declared that the whole thing was a most dangerous ‘war gas production’*” [4]. Indeed, isocyanates are typically produced by reaction of amines with phosgene [11], a highly toxic [12] gas. Alternative routes do exist at laboratory scale [11] but they still involve potentially hazardous chemicals like acyl azides, bromine or hypobromite, or even hydroxamic acids. In any case, isocyanates themselves can irritate the skin, eyes, mucous membranes, and respiratory tract, and can cause respiratory sensitization leading to occupational asthma, with possible life-threatening reactions [13]. Additional criticism has emerged after the Bhopal disaster, and the awareness of the long-term effects of isocyanates [14].

This is the reason why efforts have been made toward the production of polyurethanes through synthetic routes not involving isocyanates [15,16,17,18], while EU bodies have imposed tighter regulations [19]. These routes involve [18,20] (i) polycondensation reactions between chloroformates/carbonates and amines or carbamates/carbamoyl chlorides and alcohols, the production of which often involves phosgene, (ii) ring opening polymerization of cyclic carbamates or toxic aziridines, routes that may also involve hazardous precursors or reagents, (iii) rearrangement routes on acyl azides, carboxamides or hydroxamic acid/hydroxamate derivatives, which on one hand are hazardous themselves and on the other hand produce isocyanates in situ, (iv) transurethanization of carbamates, which avoids free isocyanates but is technologically complicated, involving removal of byproducts, control of stoichiometry, and suppression of side reactions.

A fifth route, the aminolysis of cyclic carbonates (Figure 1b), seems to be by far the most popular alternative route toward the synthesis of non-isocyanate polyurethanes [18]. From the point of view of health and safety, this route can avoid phosgene and free isocyanates and can be designed from comparatively safer precursors. Furthermore, it retains the advantages of the conventional route, i.e., the flexibility provided by the step-growth polymerization approach, and the versatility of available reactants. As with conventional polyurethanes, the process can be solvent-free. It can even be catalyst-free, as amines themselves can act as nucleophiles opening the carbonate ring [21,22]. A further, real, but often overstated, advantage is that cyclic carbonates are commonly produced by reaction of epoxy rings with CO_2_, allowing for partial incorporation of CO_2_ as a C1 feedstock into NIPU precursors [23]. Given the dominance of this approach, for the rest of the article I will deal only with this class of non-isocyanate polyurethanes, while emphasizing that structurally they are more precisely poly(hydroxy urethanes), PHUs. Unless otherwise specified, I will use the term NIPUs for these cyclic-carbonate/amine materials.

The chemistry of such systems is already well studied. The claims made so far in this manuscript are supported mostly, if not entirely, by comprehensive reviews, which is a strong sign that the synthetic side of the field is maturing. Research now seems to focus strongly on the bio-sourcing of cyclic carbonates and amines, as well as on reprocessability and recycling. This is not a NIPU-specific trend. It belongs to the broader shift in polymer technology towards renewable feedstocks, biomass valorization, and circular-economy thinking.

In the following, I will argue that a physical understanding of NIPUs is still lacking. More specifically, a predictive physical framework is needed to explain how molecular structure, thermal treatment, micromorphology, hydration, and bond exchange determine measurable macroscopic properties such as the glass transition, mechanical response, and reprocessability. I will try to point to the directions that need to be explored and propose methods to achieve this.

## 2. The Paradigm of Conventional Polyurethanes—A Solid Framework

The principles governing the properties of segmented conventional polyurethanes were largely consolidated in the early 1980s–early 1990s, with crucial contributions from Cooper, Wilkes, MacKnight, Hsu, Coleman/Painter, and of course the series of seminal quantitative works of Koberstein [10,24]. This section is not intended as a complete review of conventional polyurethanes, but as a summary of the physical paradigm that later serves as the reference point for discussing NIPUs/PHUs.

The model is elegant and simple, but the factors that affect it are numerous and often intertwined. Typical segmental polyurethanes consist at the molecular level of the so-called soft (flexible) and hard (rigid) segments. Soft segments typically originate from a polyether or any other flexible polymer terminated with OH groups. Hard segments are alternating sequences of fragments originating from a diisocyanate and a short diol, usually called a “chain extender”, which are interconnected by urethane linkages. The usual narrative is that the two types of segments are incompatible with each other and therefore they phase separate, giving a morphology of hard microdomains distributed within a soft phase. The latter consists mostly of soft segments but with some hard ones diluted in it.

For the purposes of this article, it is worth looking at the molecular origin of this incompatibility and at how it is assessed experimentally. Urethane groups, which are concentrated in the hard segments, contain both hydrogen-bond donors and acceptors. Hydrogen bonding between them is one of the main driving forces of microphase separation, while hydrogen bonding between urethane groups and the soft segments favors phase mixing. The balance between these interactions is assessed indirectly through the resulting microphase-separated morphology, for example by SAXS or WAXD, through segmental dynamics by DSC, Dielectric Spectroscopy, or DMA, or through the thermal signature of the order–disorder transition by DSC. It can also be assessed directly through the study of hydrogen bonding by FTIR. An excellent review by Yilgör, Yilgör, and Wilkes summarizes the literature up to 2015 [10] and is based on an impressive set of 208 references, showing the maturity of the field. A more methodological review, focusing on the experimental assessment of polyurethane microphase separation, was published more recently [9].

New knowledge keeps appearing, but it is out of scope of the present work. Within scope, it is necessary to mention the set of factors affecting phase separation, which will be useful for comparison with non-isocyanate polyurethanes. Those are:

- High regularity/symmetry of hard segments. This refers to both the isocyanate and the chain extender. Higher symmetry also promotes crystallinity within the hard domains [25,26]. Side chains pendent on the main chain may also have crucial effects, depending on whether they are located on the soft or the hard segments [27].
- Length of hard segments. Short hard segments, as a rule of thumb containing approximately 3–4 residual isocyanate units, tend to dissolve in the soft phase, whereas longer hard segments tend to aggregate [28,29].
- Low dispersity of hard segments. Monodisperse hard segments tend to form more stable structures.
- Content of hard segments. Higher hard-segment content tends to promote a higher degree of microphase separation.
- Low potential of the soft phase for hydrogen bonding.
- Crystallinity of both segments. This is strongly coupled with phase separation. Crystallization is favored in purer environments, and conversely formation of crystals feeds back into phase segregation [30].

Beyond chemical structure, the conditions under which phase separation was formed should never be overlooked. Thermal history, in terms of annealing temperature or cooling rate from the melt, is particularly important because it can significantly modify the degree of phase separation, as well as the internal ordering of the domains [31]. A more recent work also identifies a mesophase separation in the length scale of μm, which harbors the traditionally understood one, and may persist at temperatures above the hard segment melting [32]. The effect of the synthesis route, including different solvents or solvent-free conditions, has been explored in the past and is still being considered [33,34]. Another recent work highlights the influence of solvent in solvent vapor annealing [35].

It can be stated that microphase separation is the bridge between molecular design and actual macroscopic performance. Strong microphase separation is known to increase Young’s modulus in the rubbery plateau and mechanical strength. In strongly separated systems, the glass-transition temperature associated with the soft phase is low, approaching that of the pure polyol, usually well below room temperature, whereas in phase-mixed systems it increases according to the mixing laws governing polymer mixtures and copolymers [10,36,37]. Above room temperature, the thermal behavior of polyurethanes is dominated by the properties of hard microdomains. Below 100 °C, weak effects associated with the relaxation of hard microdomains may occur. In the reprocessing-relevant region between 100 and 200 °C, more intense thermal events may appear, including amorphous hard-domain disordering, microphase mixing, melting of crystalline hard domains, and possible recrystallization [31,36]. In practice, microphase separation can be controlled by molecular design through hard-segment content and regularity, soft-segment polarity and molar mass, chain-extender structure, or use of crystallizable segments. Beyond molecular design it can be influenced by proper choice of solvent and evaporation rate during synthesis or processing. Post-synthesis, however, the most accessible way to control microphase separation is the proper selection of thermal history during processing. The desired degree of microphase separation depends on the application: stronger phase separation generally favors modulus, strength, elastic recovery, dimensional stability, and lower water uptake, whereas weaker phase separation may be useful for damping, adhesion, and transparency.

When it comes to water absorption, high microphase separation is often associated with lower water absorption capacity. It is generally accepted that the main hydration sites in conventional polyurethanes are, in approximate order of importance: (i) urethane carbonyls, especially free or disordered ones; (ii) urethane N–H groups as hydrogen-bond donors; (iii) ester carbonyls in polyester soft segments; and (iv) ether oxygens in polyether soft segments, especially PEG/PEO [38]. In general, high microphase separation tends to decrease water absorption and thus increase hydrolytic stability [39], although exceptions related to a highly hydrophilic soft phase exposed to liquid water may give the opposite effect [40]. In any case, there is evidence that microphase separation tends to inhibit water absorption [41], an observation that should be attributed to limited accessibility of water to the primary hydration sites on the urethane groups. Competition for hydrogen bonding at carbamate groups favors other urethane units rather than H_2_O [42], and only minimal plasticization or morphological change of hard domains is observed on the time scale of approximately 30 h. Highly hydrophilic, non-segmental, polyurethane networks can form hydrogels with water contents corresponding to approximately 1000 wt% water relative to dry polymer upon immersion in water [43]. To my knowledge, comparable water-vapor sorption data remain scarce, although such data would help clarify the hydration process under realistic service conditions. In the case of PEG, humidity may induce a transition in which semicrystalline soft segments switch to a gel-like morphology above a certain relative humidity level [44].

**Figure 2:**
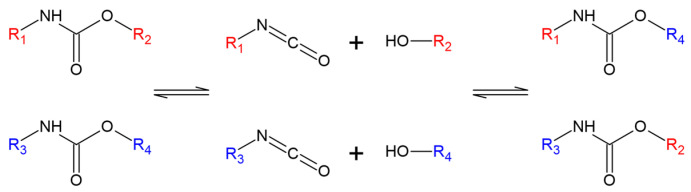
Urethane exchange reactions in conventional polyurethanes through dissociation to isocyanate and alcohol groups followed by reassociation.

A last comment regarding conventional polyurethanes is that, in a basic stoichiometric configuration, the material is usually considered chemically rather stable after synthesis. In a basic stoichiometric configuration, the only realistic reconfiguration of chains is through the dissociative mechanism of urethane exchange, that is, dissociation of two urethane groups to NCO and OH and reassociation (Figure 2). This process is a very slow one, and depends a lot on temperature, chemical nature of components and the presence of any catalysts [45]. Recently, with an emerging interest in vitrimeric materials, polyurethanes have been specifically designed to provide dynamic changes in the network topology. However, this requires either non-stoichiometric approaches or introduction of other functionalities on one of the components. For example, dynamic change of topology has been achieved by an excess of isocyanate groups, and exploiting the reversible crosslinking reactions they can provide, namely formation of either uretdiones or allophanates, and in a second step isocyanurate rings [46]. A more elaborate approach is by introduction of other functionalities on one of the components, e.g., a secondary amine on the chain extender, which gave rise to reversible crosslinking by hindered urea bonds [47]. 

In the following sections, I will discuss how the concepts of phase separation, water absorption, and chain reconfiguration change in NIPUs/PHUs obtained by polyaddition of cyclic carbonates and amines. These phenomena are directly relevant to macroscopic properties such as mechanical performance, thermal transport, and, in porous systems, pore-size distribution. A detailed discussion of each application-specific property is beyond the scope of this perspective; instead, the emphasis is placed on the underlying physical framework from which these properties emerge.

## 3. Microphase Separation in NIPUs/PHUs

The hydroxyl group produced by the aminolysis of cyclic carbonates, alongside the urethane group, can strongly hinder microphase separation, at least in the conventional sense. There are a few fundamental reasons for this. (i) As pendent groups, they may sterically hinder hydrogen bonding between urethane units; (ii) they introduce irregular stereochemistry and regiochemistry, because cyclic-carbonate aminolysis often gives mixtures of primary- and secondary-hydroxyl structures, thus breaking the periodicity of hard segments; (iii) they form hydrogen bonds of their own, especially with the soft phase, which more often than not contains polar ether or ester units.

Feedstock selection also imposes a practical, although not fundamental, restriction on phase separation. Research in the field is largely driven by the exploitation of renewable resources, such as vegetable oils [48,49], terpenes [50], lignin derivatives [51,52], sugar alcohols [53,54] and other bioderived polyols [55]. These feedstocks are attractive from the sustainability perspective and for applications such as coatings and adhesives. However, they are often multifunctional, asymmetric, and conformationally or structurally irregular. These traits work against the architectural regularity, linearity, and hard segment packing needed for classical microphase separation. It should be stressed, however, that renewable origin does not inherently imply poor microphase separation, since bio-based precursors can also be regular and can give structured morphologies [54].

In fact, non-isocyanate polyurethanes can give microphase-separated structures. The critical role of hydroxyl groups in phase separation was noticed early, although in a system that also contained classical urethane units [56]. In an early work with segmented poly(hydroxy urethanes), phase separation was reported for a system based on poly(tetramethylene oxide) (PTMO) with 50 and 70 wt% aromatic hard segments. At 30 wt% hard segments the material is phase-mixed [57]. Soon afterwards, Torkelson’s group showed that the soft segment is crucial for the development of microphase separation [58]. Hydroxyurethane units formed hydrogen bonds with poly(ethylene oxide) soft segments, driving phase mixing, which resulted in viscous, flowing materials. This is in contrast to an analogous conventional system, which gave excellent mechanical properties. Poly(propylene oxide) (PPO) and PTMO gave phase-separated materials at 40–50 wt% of the hard phase. Poly(dimethyl siloxane), especially of low molar mass, also favors phase separation [59]. A later study confirms good phase separation in PTMO [60]. The same study reports mechanically stable materials based on other relatively long polyethers. However, this is not by itself secure evidence of microphase separation, because the mechanical stability could also arise from extensive hydrogen bonding in the system [61].

Within these PTMO/aromatic hard-segment systems, the nature of the chain extender does not seem to be the dominant parameter. NIPUs based on PTMO of Mw ≈ 1700 and divinyl benzene dicyclocarbonate with seven structurally different amines as chain extenders were all phase-separated at reasonable hard-segment contents down to 30 wt% [62,63]. Functionalization of OH groups with acetic anhydride completely suppressed microphase separation, presumably as a result of steric hindrance by the bulky groups [64].

Interestingly, phase separation can even occur in systems without conventional linear hard segments, for example when a long linear diamine is crosslinked with a trifunctional cyclic carbonate. In this case, PTMO of Mn ≈ 1600 did not phase separate, whereas polybutadiene (PB, Mn ≈ 3800) and polydimethylsiloxane (PDMS, Mn ≈ 900) did [65]. This is consistent with the lower polarity of PB and PDMS and the weaker competition between soft-segment hydrogen bonding and hydroxyurethane association. Not surprisingly, the microphase separation could be tuned with the addition of chain extenders [66]. When the chain extender was a diamine, and thus located closer to the network nodes, it had a more prominent effect, giving microphase separation even in the PTMO network. This can be attributed to the higher concentration of urethane units around the nodes, even if they are not able to pack in a fully conventional hard-domain structure.

In an alternative approach, microphase separation may be driven, or at least strongly favored, by crystallinity of chains between the urethane units [67,68]. This was nicely demonstrated in a system containing hard segments based on diaminododecane as a chain extender and using 4,9-dioxa-1,12-dodecanediamine as a spacer in the mainly PEO-based soft phase [67]. This approach was exploited very recently by Hebda et al. [54] to produce ordered systems based on hard segments made of 1,12-diaminododecane and erythritol decarbonate and soft segments consisting of relatively long chains (Mn ≈ 2000) of various polyethers, namely PEO, PPO, and a PPO–PEO–PPO triblock. All systems were phase-separated, albeit the mass fractions of the hard domains were more than 70 wt%. The nature of phase separation was different in each case: In the PPO environment, it seemed to be driven by ordering in the hard domains, which is compatible with the earlier knowledge that PPO, an amorphous polymer, is unfavorable for hydrogen bonding of the hydroxyls. On the contrary, in the PEO environment and the copolymer the phase separation seems to be driven by the crystallinity of the rather long PEO chains. This is corroborated by a similar work by Li et al. [69]. Apparently, EO–EO association may become more favorable than EO–OH association under these conditions.

More recently, strongly phase-separated materials were produced from biobased, yet well-defined, resources [70,71]. According to the authors, the success was mostly due to lack of intersegmental hydrogen bonding [70].

Thus, despite the less favorable environment for classical hard-domain formation, NIPUs/PHUs can and do attain microphase-separated morphologies. Nevertheless, the nature of the phase separation, and especially its kinetics, are not sufficiently studied. At first glance this is counterintuitive. After all, it is well established that phase separation in conventional polyurethanes can be tuned by appropriate control of thermal history [31,72]. The physical processes in conventional PUs were understood by temperature-resolved studies, including temperature-resolved FTIR, WAXD, SAXS, and the simpler, yet very effective, DSC. Yet, such studies remain scarce for NIPUs/PHUs. I am only aware of a work by Zhang et al. [71], which studied a polymer based on aminolysis of both cyclic carbonates and esters. Interestingly, none of the works mentioned above studies in detail the thermal transitions associated with phase separation, although DSC curves are often shown. A possible exception is the work by Hebda et al. [54], which however comments mainly on melting transitions at low temperatures, whereas the order–disorder transition associated with hydrogen bonding between urethanes is known to occur at temperatures close to 200 °C.

In the next section, we will see that one reason for this gap is that, at temperatures relevant for reprocessing polyurethanes, bond-exchange reactions are activated in NIPUs/PHUs and complicate the interpretation of the dynamics. Therefore, reports on microphase separation usually concern as-prepared or as-cast samples, while systematic thermal control remains rare. Nevertheless, several parameters still provide opportunities for tailoring microphase separation. Among them we should note the chemical nature of the solvent, if any, the viscosity of the system, the rate of evaporation, the temperature and pressure during film formation, and of course the structure of the polyurethane itself [73,74]. The literature regarding these parameters is already scarce in conventional polyurethanes, and I was not able to find relevant works for NIPUs. Nevertheless, given the complicated landscape of thermal treatment at reprocessing temperatures, controlling microphase separation during synthesis or by solvent casting might be a simple and effective approach.

## 4. Reprocessing NIPUs: Chemistry Landscape Above 100 °C

While phase separation is still poorly studied, the concept of reprocessing NIPUs is studied extensively. In many segmented thermoplastic conventional PUs, reprocessing is achieved by disordering or melting the hard microdomains, which act as physical crosslinks, giving essentially a reprocessable melt. This typically happens at temperatures close to 180 °C, although the exact temperature depends strongly on structure. In NIPUs/PHUs, before this point is reached, several bond-exchange reactions may take place, changing chain connectivity and, in networks, topology. These reactions are dynamic and reversible, and they may allow easier reprocessing at lower temperatures. This advantage, however, comes with a design constraint: bond exchange and segmental mobility should be limited under service conditions and become significant mainly under reprocessing conditions. Mechanical integrity therefore depends on persistent constraints such as chemical crosslinks, microphase-separated hard domains, crystallizable segments, or strong inter-chain hydrogen bonding.

**Figure 3:**
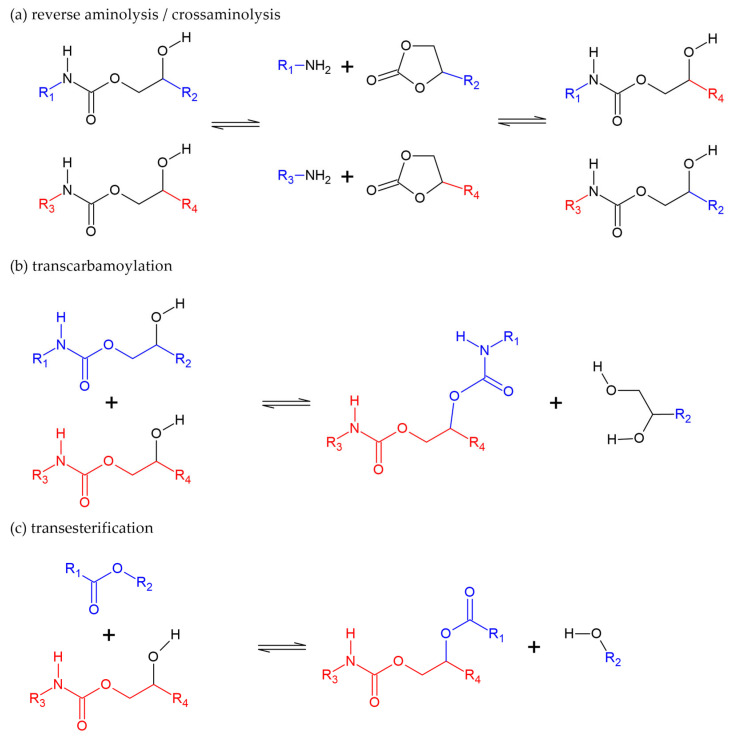
Bond-exchange reactions activated at elevated temperatures in NIPUs/PHUs: (**a**) reversible aminolysis leading to cyclic carbonate and amine groups; (**b**) transcarbamoylation involving reaction of a hydroxyl group with a urethane carbonyl; and (**c**) transesterification when esters are present in the chain.

The first key reaction is reversible aminolysis, which is essentially a depolymerization process (Figure 3a) [75]. Scission of the hydroxyurethane unit can regenerate an amine and a cyclic carbonate. These newly formed reactive groups may react with other units, enabling dynamic exchange of bonds.

The second key reaction is transcarbamoylation [45,76] (Figure 3b). This reaction is intensively used in the chemical recycling of conventional polyurethanes by glycolysis [77,78,79], and in NIPUs it can be regarded as a built-in feature. In the case of NIPUs/PHUs, a hydroxyl group of one hydroxyurethane unit attacks the carbamate carbonyl of a nearby urethane segment, resulting in exchange of the alkoxy substituent on the urethane. This forms a new urethane linkage at the attacking hydroxyl site and liberates the former O-side of the original hydroxyurethane as an alcohol-bearing fragment. If the attacked unit contains a free β-hydroxyl, the released fragment is diol-like, and may further participate in bond-exchange reactions.

Beyond the intrinsic bond-exchange reactions of hydroxyurethanes, the system may also contain other functional groups capable of exchange. A nice example is the ester groups introduced in a heavily crosslinked system by Hu et al. [75]. These groups can undergo transesterification reactions with NIPU hydroxyls, in an effect analogous to transcarbamoylation (Figure 3c).

These dynamic molecular rearrangements are activated in the temperature region relevant for reprocessing, roughly 100–200 °C, and may occur concurrently. In addition, they can be controlled by network structure, catalysts, and acidity of the system [80,81,82,83,84,85,86]. The dynamics of the processes is usually discussed in terms of macroscopic stress relaxation/reprocessing time scales. The relaxation times reported in the typical reprocessing temperature window fall in the experimentally accessible range of 10^2^–10^4^ s, that is, from minutes to hours [80,81,83,84,86]. This macroscopic, technologically relevant time scale should be understood as an apparent response of the material arising from coupled chemical exchange and network dynamics. It should be governed by the intrinsic exchange kinetics, the concentration and the accessibility of reactive groups. The latter, however, in these highly viscous systems is largely governed by segmental dynamics and inter-chain diffusion. This is a special case of the broader vitrimer context, where bond-exchange kinetics and segmental dynamics are strongly coupled, especially when their time scales become comparable [87,88]. The landscape becomes even more complicated when NIPUs/PHUs are segmented, because microphase separation will also affect the macroscopic kinetics of reprocessability. It should also be noted that the same chemistry that enables reprocessing may also lead to property drift during prolonged thermal exposure, or repeated reprocessing rounds. Thus aging, creep, and dimensional stability should be considered together with reprocessability.

Summing up, from the technological point of view, NIPUs can show useful mechanical properties, and can be intrinsically reprocessable, whether they are segmented and phase-separated or not. In crosslinked systems, this can be achieved in a vitrimer-like approach, where chemical crosslinking provides dimensional stability while dynamic bonds allow reprocessing, even if the crosslink density does not decrease with temperature. Interestingly, even linear, unstructured, amorphous systems can show good mechanical stability due to inter-chain hydrogen bonding, either between hydroxy-urethanes or between hydroxyurethanes and polar groups on soft segments [61]. If, in addition, the system is segmented, traditional phase separation can develop and play a significant role in the macroscopic properties, analogously to conventional polyurethanes.

So far, the literature reports extensively on the technological success of several NIPU/PHU designs, especially with respect to mechanical properties and reprocessability. The concept and the feasibility of the approach are well justified. However, a microscopic and molecular-level understanding is still underdeveloped largely because hydrogen bonding, morphology, segmental mobility, and bond exchange are strongly coupled and difficult to separate experimentally. A further limitation is the absence of a theoretical framework that treats these processes together rather than as independent contributions to macroscopic behavior. Routine macroscopic methods cannot decouple these contributions. It is reasonable to assume that the slowest process will dominate the macroscopic dynamics. However, if the time scales are comparable, each process may affect the others considerably. Changes in the degree of polymerization, crosslinking density or topology will affect the segmental dynamics and viscosity of the system. Conversely, the molecular dynamics will affect the rate by which bond-exchange reaction will take place.

A deeper understanding of these intertwined phenomena, particularly how they influence one another, is needed to move beyond trial-and-error formulation toward informed, predictive design. Such understanding would enable PHU/NIPU materials to be tailored for targeted properties while preserving reprocessability and recyclability. Furthermore, and perhaps more importantly, it will allow for tailoring properties by controlling thermal history. This is particularly attractive in the context of 3D printing, where one may imagine printing parts with graded properties using a single-material filament [89].

The development of the conventional PU paradigm described earlier was largely achieved by Differential Scanning Calorimetry (DSC) and temperature-resolved Small-Angle X-ray Scattering (SAXS), often applied simultaneously. Infrared Spectroscopy gave an understanding of the hydrogen bonding at the molecular level. Segmental dynamics were effectively studied by Dynamic Mechanical Analysis (DMA) and Broadband Dielectric Relaxation Spectroscopy (DRS). In order to develop an equally solid paradigm for NIPUs, we need to look at the molecular level and its dynamics in tandem with the macroscopic properties. In other words, we need to measure several length and time scales simultaneously in order to disentangle, as far as possible, the contributions of chemical reactions, molecular mobility, and morphology.

A reasonable strategy is to monitor the evolution of selected molecular and segmental observables after a controlled perturbation, for example a temperature jump. These would then be compared with the time scale of macroscopic relaxation or reprocessing. An example would be monitoring segmental dynamics by recording consecutive dielectric spectra over the time range of macroscopic equilibration immediately after a stepwise change in temperature [90]. However, at elevated temperatures traditional dielectric, mechanical, or rheological measurements may not be sufficient. The reason is that the time scales of segmental dynamics may move outside the accessible range of these techniques. In addition, the acquisition time of a full spectrum may be too long to monitor its evolution with time. Dielectric spectroscopy in particular might also be limited by high conductivity at elevated temperatures. Thus, complementary methods for the study of local and collective dynamics may be needed. Quasi-elastic neutron scattering (QENS) can access Å to nm motions in ps to ns time scales [91], while Neutron Spin Echo (NSE) can access nm and ns scales [92]. These methods would not observe elementary bond-exchange rates directly, but they would reveal whether changes in mobility correlate with reprocessing rate.

If the aforementioned methods are coupled with, or at least compared with, solid-state NMR, the time scale of exchange reactions may be monitored more directly, provided that the exchanging species are spectroscopically distinguishable. Such measurements would allow the rate of chemical exchange to be studied in conjunction with segmental dynamics. If phase separation is expected, simultaneous or even off-line scattering experiments would also be useful for monitoring changes in the nano-scale.

It is worth mentioning that a net change in the chemical equilibrium of the bond-exchange reactions upon a thermal perturbation is not required for reprocessability. In case the equilibrium does not shift, or shifts very weakly, following the time scale becomes non-trivial, because there would be no net change in spectral lines, e.g., in NMR, or in heat flow in DSC experiments. In principle, isotope labeling might give some information on the matter [93,94] but such experiments remain non-trivial in polymer science. Indirect strategies may therefore be needed, such as systematic variation of hydroxyl density, catalyst concentration, acidity, or soft-segment polarity, followed by comparison of exchange-sensitive and mobility-sensitive observables.

## 5. The Glass Transition of NIPUs/PHUs and Its Relation to Humidity

At first glance, the glass transition of NIPUs/PHUs appears comparatively conventional. Interesting issues, however, arise when hydration is considered.

Glass transition in non-segmental linear systems comprising polyether segments originating from bis(cyclic carbonates) interconnected with fragments originating from amines follows the normal principles expected in polymers. The dynamics is mostly dominated by the polyether, but it is slowed down to different extents depending on the bulkiness/flexibility of the amine fragment, as well as their concentration in the system, and the molar mass of the polyether fragments [95]. The cooperativity seems to depend only on the polyether, unless the amine-originating fragment forms stronger hydrogen bonding with nearby segments [95]. Interestingly, however, in a system based on PPO with molar mass 700, a deviation from the low-temperature Vogel Fulcher Tammann behavior occurs around 90 °C, the origin of which is not clarified at this point. It is worth studying whether this deviation is related to the activation of bond-exchange reactions, as described earlier.

In a similar poly(ethylene oxide) (PEO)-based system, interconnected with two types of amines at various ratios, and with limited hydrogen bonding, the glass transition temperature (*T_g_*) depends on the type of amine, and for intermediate compositions *T_g_* follows the Couchman–Karasz model [96]. Contrary to that, in a similar system based on poly(propylene oxide) (PPO) with shorter chains, the intersegmental hydrogen bonding plays a significant role, and largely dominates the mobility by slowing down the dynamics, and defines the mechanical properties [61].

In hybrid organic–inorganic NIPU-POSS systems, with POSS either substituting network nodes or terminating the chains, the molecular dynamics also shows the expected behavior. The bulky POSS molecules give rise to decelerated dynamics compatible with the formation of rigid amorphous fraction, while disrupting the network-node density accelerates the dynamics [97,98]. In another network, the efficiency of the reaction, which depends on the employed catalyst, also has an impact on *T_g_*, while slight inefficiency in full reprocessing causes a slight decrease in *T_g_* [86].

As expected, systems based on low-molecular-mass components give quite high glass-transition temperatures [99] comparable to those of similarly structured conventional polyurethane systems [100]. In analogy with conventional PUs, and in general microphase-separated copolymers, microphase-separated NIPUs exhibit two glass transitions [54,70].

There is, however, an indirect particularity in the glass transition of NIPUs, and it is related to their hydrophilicity. Pendent hydroxyls constitute hydration sites. In addition, weaker microphase separation may allow more urethane units, which are also hydration sites, to remain accessible to water molecules. These effects combined render NIPUs, especially when non-segmental or non-phase-separated, very hydrophilic. A few detailed studies on the hydration of NIPUs/PHUs have been conducted so far. Even systems based on hydrophobic PPO can absorb up to 80 wt% with respect to dry mass when exposed to relative humidity *rh* 98%, depending also on the hydrophilicity of the amine component and the competition with intersegmental hydrogen bonding [101]. The water-sorption isotherms of these systems, that is, water uptake as a function of relative humidity, are type III in the Brunauer classification, typical of soluble systems [102]. As expected, this water accelerates the segmental dynamics of the system. Interestingly, there seem to be three distinct regions in this effect. This, in combination with FTIR spectra, can be discussed in terms of the following model. First, water molecules attack to some extent the intersegmental hydrogen bonds, releasing constraints and lowering *T_g_*. Next, water molecules distribute along the chain, weakening intra-chain interactions and increasing chain flexibility, a process referred to as “plasticization”. This second regime seems centered around the water content corresponding to monolayer capacity, that is, the amount of water that can be directly bound on hydration sites along the chain. In the third regime, starting when there is enough semi-bound or free water, the dynamics of the system become collective and reflect a combination of polymer dynamics and the much faster dynamics of free water, a phenomenon often described as “slaving”. The second and third regimes are very well described collectively by the Kwei mixing law [103], characteristic of interacting blended molecules. In a less detailed study with an analogous system based on PEO, despite the higher hydrophilicity of the polyether, the water absorption values are similar to the PPO system, highlighting the hydroxyurethane units as the primary hydration sites [96]. It is also interesting that, in the third regime, in both cases the details of the amine component do not have any marked effect on *T_g_* and the dominant parameters are the nature of the polyether and the water content. This further corroborates the assumption that chain–chain interactions, which are compromised at high hydrations, are crucial in the dry systems.

NIPU/PHU networks based on these common polyethers are also highly hydrophilic, reaching water uptakes over 100 wt% for PEO-based systems [104] and 60 wt% for PPO-based systems [97]. As with the linear systems, there seem to be two distinct regions in the effect of water on the glass transition, discussed in terms of plasticization and slaving.

These effects are crucial for applications and for understanding the dynamics. It is worth stressing that the glass-transition temperature can drop by several tens of kelvin between the dry material and the material equilibrated at 50% relative humidity, which is a realistic humidity value for environments where polyurethanes are expected to operate.

A caveat is that these studies were conducted in largely phase-mixed systems, which are not directly comparable with conventional segmental polyurethanes. As discussed earlier, phase separation seems to inhibit water absorption and increase hydrolytic stability [39,40,41]. Therefore, hydration studies should also be conducted in segmented NIPU/PHU systems. Particularly useful experiments would include humidity-controlled DMA, DRS, FTIR, and SAXS/WAXD, so that changes in glass transition, hydrogen bonding, and morphology can be followed under the same hydration conditions.

Whether absorbed water only plasticizes the material or also accelerates chemical degradation will depend on the presence of hydrolysable groups, the accessibility of urethane/ester bonds, and the degree of phase separation. These factors will also likely have an indirect impact on glass transition, and should be taken into account during experimental study.

## 6. Outlook

NIPUs/PHUs synthesized by aminolysis of cyclic carbonates open a fundamentally new chapter in the context of polymers prepared by step-growth polymerization in general and polyurethanes specifically. On one hand, the pendent hydroxyl in their structure gives them some of the attractive features of vitrimers, namely the possibility of combining crosslinking with reprocessability through bond-exchange reactions. On the other hand, NIPUs/PHUs remain polyurethanes, and a segmented structure can still provide microphase separation and thus the possibility of tuning physical properties by proper thermal conditioning. Therefore, it becomes a matter of molecular design to choose which of these two characters will prevail in the final material, and how their combination can provide new functional properties.

For an informed design, it is important to develop a predictive model which will connect the final properties with structure and prior conditioning. Prerequisite for such a framework is to understand the mechanisms and time scales of bond-exchange reactions, molecular mobility, and development of micromorphology. This is a non-trivial endeavor. The working model for conventional polyurethanes is a useful starting point, but it needs to be expanded with a model for the extent and time scale of bond exchange, which is intimately connected with the extent and time scale of micromorphology development. Molecular dynamics is one of the connectors between them. In order to disentangle these phenomena, the experimental toolkit must also be expanded with methods that directly probe the molecular level over broad time- and length-scale regimes.

So far, Chemistry and Chemical Technology have driven the growth of the NIPU/PHU field. Works focusing on fundamental chemistry are published with an increasing rate, alongside works focusing on bio-sourcing. These studies so far have provided convincing proof of concept for reprocessability and for the other good properties expected from NIPUs/PHUs. The field is now mature enough for tools from Polymer Physics, both theoretical and experimental, to be employed more systematically. There is, however, an important distinction: the new physical models will need to take into account that the molecular architecture is no longer a constant variable, but changes dynamically.

**Figure 4:**
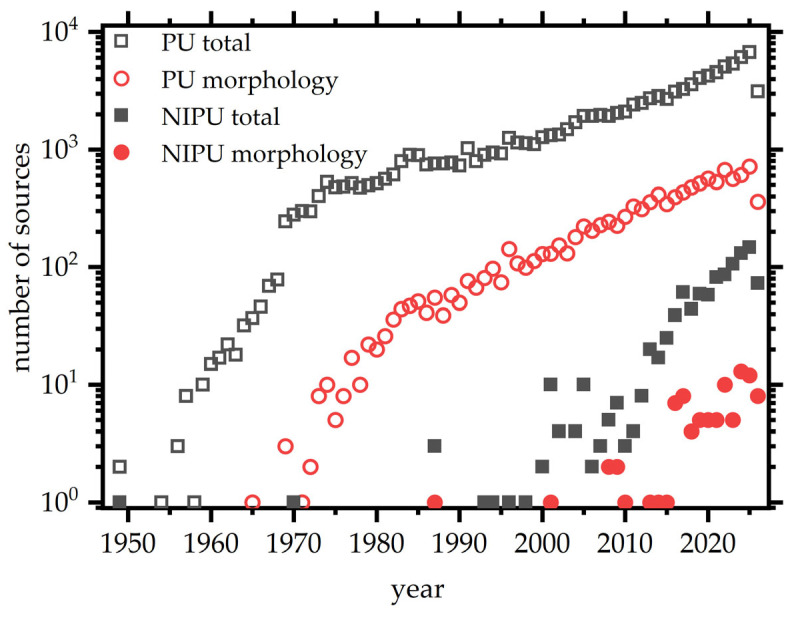
Indicative Scopus source counts for polyurethane and NIPU/PHU publications, with and without morphology-related search terms. Searches were performed on 19 May 2026 using TITLE-ABS-KEY fields. Search strings are given in Appendix A. The counts should be read as indicative rather than exhaustive.

The imbalance is illustrated, in a crude bibliometric sense, in Figure 4. The NIPU/PHU literature has expanded rapidly, but the subset of papers explicitly addressing microphase separation, nanophase separation, morphology, or microdomains remains small compared with the broader NIPU/PHU literature. The same qualitative delay existed historically in conventional polyurethanes: synthetic and technological development preceded the consolidation of the physical morphology–property framework. Although Figure 4 is not meant as a means for quantitative prediction, it indicates that morphology-focused physical studies of NIPUs/PHUs are still at an early stage. Hopefully, the synergy between chemistry and physics may provide a concrete understanding of NIPUs/PHUs within the next decade.

The central challenge at this point is not simply to make NIPUs greener or more reprocessable, but to understand what kind of polymer physics they make possible. NIPUs/PHUs are not a simple extension of conventional polyurethanes. The pendent hydroxyl transforms the urethane linkage from a familiar structural motif into a source of competing hydrogen bonds, hydration sites, and thermally activated exchange reactions. This makes NIPUs/PHUs harder to describe using existing PU concepts, but also opens a wider design space: materials whose architecture, morphology, and dynamics can be deliberately coupled during processing and use. The next step is to turn this complexity into predictive design.

## Data Availability

No original experimental data were generated in this study. The bibliometric data supporting Figure 4 consist of Scopus source counts obtained using the search strings and filters reported in Appendix A. These data are derived from a subscription bibliographic database and may vary depending on the date of access and database coverage. Further inquiries can be directed to the author.

## Appendix A

### Data in Figure 4

Figure 4 is based on Scopus source counts. The searches were performed on 19 May 2026 using the Scopus Advanced Search tool. The search field was restricted to TITLE-ABS-KEY. No additional filters were applied.

The keys used were:

- Query “PU total”: TITLE-ABS-KEY (polyurethane OR polyurethanes OR “poly(urethane)” OR “poly(urethanes)”).
- Query “PU morphology”: TITLE-ABS-KEY ((polyurethane OR polyurethanes OR “poly(urethane)” OR “poly(urethanes)”) AND (“microphase separation” OR “micro-phase separation” OR “phase separation” OR “nanophase separation” OR “nano-phase separation” OR morphology OR microdomain OR microdomains)).
- Query “NIPU total”: TITLE-ABS-KEY (“non-isocyanate polyurethane” OR “non isocyanate polyurethane” OR “nonisocyanate polyurethane” OR NIPU OR NIPUs OR polyhydroxyurethane OR polyhydroxyurethanes OR “poly(hydroxyurethane)” OR “poly(hydroxyurethane)s” OR “poly(hydroxy urethane)” OR “poly(hydroxy urethane)s”).
- Query “NIPU morphology”: TITLE-ABS-KEY ((“non-isocyanate polyurethane” OR “non isocyanate polyurethane” OR “nonisocyanate polyurethane” OR NIPU OR NIPUs OR polyhydroxyurethane OR polyhydroxyurethanes OR “poly(hydroxyurethane)” OR “poly(hydroxyurethane)s” OR “poly(hydroxy urethane)” OR “poly(hydroxy urethane)s”) AND (“microphase separation” OR “micro-phase separation” OR “phase separation” OR “nanophase separation” OR “nano-phase separation” OR morphology OR microdomain OR microdomains)).

## References

[1] Carothers W.H. (1929). Studies on Polymerization and Ring Formation. I. An Introduction to the General Theory of Condensation Polymers. J. Am. Chem. Soc..

[2] Viswanathan A. (2010). Wallace Carothers: More than the Inventor of Nylon and Neoprene. World Pat. Inf..

[3] Carothers W.H. (1931). Polymerization. Chem. Rev..

[4] Bayer O. (1975). The Odyssey of an Invention Charles Goodyear Medal Address—1975. Rubber Chem. Technol..

[5] IG Farbenindustrie AG (1942). Process for the Production of Polyurethanes or Polyureas.

[6] Delavarde A., Savin G., Derkenne P., Boursier M., Morales-Cerrada R., Nottelet B., Pinaud J., Caillol S. (2024). Sustainable Polyurethanes: Toward New Cutting-Edge Opportunities. Prog. Polym. Sci..

[7] Wu S., Ma S., Zhang Q., Yang C. (2025). A Comprehensive Review of Polyurethane: Properties, Applications and Future Perspectives. Polymer.

[8] Rossignolo G., Malucelli G., Lorenzetti A. (2024). Recycling of Polyurethanes: Where We Are and Where We Are Going. Green Chem..

[9] Cheng B.X., Gao W.C., Ren X.M., Ouyang X.Y., Zhao Y., Zhao H., Wu W., Huang C.X., Liu Y., Liu X.Y. (2022). A Review of Microphase Separation of Polyurethane: Characterization and Applications. Polym. Test..

[10] Yilgör I., Yilgör E., Wilkes G.L. (2015). Critical Parameters in Designing Segmented Polyurethanes and Their Effect on Morphology and Properties: A Comprehensive Review. Polymer.

[11] Niesiobędzka J., Datta J. (2023). Challenges and Recent Advances in Bio-Based Isocyanate Production. Green Chem..

[12] Pauluhn J. (2021). Phosgene Inhalation Toxicity: Update on Mechanisms and Mechanism-Based Treatment Strategies. Toxicology.

[13] Reh B.D. (2004). Summary of Health Hazard Evaluations: Issues Related to Occupational Exposure to Isocyanates, 1989 to 2002.

[14] Dhara V.R., Dhara R. (2002). The Union Carbide Disaster in Bhopal: A Review of Health Effects. Arch. Environ. Health Int. J..

[15] Balla E., Bikiaris D.N., Pardalis N., Bikiaris N.D. (2025). Toward Sustainable Polyurethane Alternatives: A Review of the Synthesis, Applications, and Lifecycle of Non-Isocyanate Polyurethanes (NIPUs). Polymers.

[16] Rokicki G., Parzuchowski P.G., Mazurek M. (2015). Non-Isocyanate Polyurethanes: Synthesis, Properties, and Applications. Polym. Adv. Technol..

[17] Khatoon H., Iqbal S., Irfan M., Darda A., Rawat N.K. (2021). A Review on the Production, Properties and Applications of Non-Isocyanate Polyurethane: A Greener Perspective. Prog. Org. Coat..

[18] Rayung M., Ghani N.A., Hasanudin N. (2024). A Review on Vegetable Oil-Based Non Isocyanate Polyurethane: Towards a Greener and Sustainable Production Route. RSC Adv..

[19] Commission Regulation (EU)—2020/1149. https://eur-lex.europa.eu/eli/reg/2020/1149/oj/eng.

[20] Orabona F., Recupido F., Lama G.C., Polaczek K., Taddeo F., Salmi T., Di Serio M., Verdolotti L., Russo V. (2025). Cutting-Edge Development of Non-Isocyanate Polyurethane (NIPU) Foams: From Sustainable Precursors to Environmental Impact Evaluation. Green Chem..

[21] Gaida R., Kiełkiewicz D., Biernat N., Furgoł S., Dudek G. (2026). Optimizing Poly(Hydroxyurethane) Synthesis via Cyclic Carbonate Aminolysis: A Comprehensive Review. Green Chem..

[22] Lambeth R.H., Henderson T.J. (2013). Organocatalytic Synthesis of (Poly)Hydroxyurethanes from Cyclic Carbonates and Amines. Polymer.

[23] Guo L., Lamb K.J., North M. (2021). Recent Developments in Organocatalysed Transformations of Epoxides and Carbon Dioxide into Cyclic Carbonates. Green Chem..

[24] Prisacariu C. (2011). Polyurethane Elastomers.

[25] Tian Y., Wang M., Wang J., Wei Y., Chang Y., Qin X., Zhang L. (2026). Tuning Hard-Segment Regularity for Microphase Control and Property Enhancement in Hydroxyl-Terminated Polybutadiene-Based Polyurethane Elastomers. Macromolecules.

[26] Klinedinst D.B., Yilgör I., Yilgör E., Zhang M., Wilkes G.L. (2012). The Effect of Varying Soft and Hard Segment Length on the Structure–Property Relationships of Segmented Polyurethanes Based on a Linear Symmetric Diisocyanate, 1,4-Butanediol and PTMO Soft Segments. Polymer.

[27] Tantisuwanno C., Jain T., Tseng Y.M., Joy A. (2024). Pendant Amines in the Hard or Soft Segments of PCL-Polyurethanes Have Contrasting Effects on the Mechanical and Surface Properties. Macromolecules.

[28] Koberstein J.T., Russell T.P. (1986). Simultaneous SAXS-DSC Study of Multiple Endothermic Behavior in Polyether-Based Polyurethane Block Copolymers. Macromolecules.

[29] Koberstein J.T., Leung L.M. (1992). Compression-Molded Polyurethane Block Copolymers. 2. Evaluation of Microphase Compositions. Macromolecules.

[30] Korley L.S.T.J., Pate B.D., Thomas E.L., Hammond P.T. (2006). Effect of the Degree of Soft and Hard Segment Ordering on the Morphology and Mechanical Behavior of Semicrystalline Segmented Polyurethanes. Polymer.

[31] Szefer E., Stafin K., Leszczyńska A., Zając P., Hebda E., Raftopoulos K.N., Pielichowski K. (2019). Morphology, Dynamics, and Order Development in a Thermoplastic Polyurethane with Melt Blended POSS. J. Polym. Sci. Part B Polym. Phys..

[32] Lee Y.-C., Lin W.-C., Zhang Y.-Q., Hua C.-C. (2026). Parallel Micro- and Mesophase Transitions of Thermoplastic Polyurethane Induced by Thermal Annealing. Soft Matter.

[33] Ramezani M., Getya D., Gitsov I., Monroe M.B.B. (2024). Solvent-Free Synthesis of Biostable Segmented Polyurethane Shape Memory Polymers for Biomedical Applications. J. Mater. Chem. B.

[34] Yoon S.C., Ratner B.D. (1988). Surface and Bulk Structure of Segmented Poly(Ether Urethanes) with Perfluoro Chain Extenders. 3. Effects of Annealing, Casting Solvent, and Casting Conditions. Macromolecules.

[35] Wang S., Song J., Chen K., Henderson M.J., Tian Q., Almásy L. (2025). Solvent-Annealing-Induced Microphase Separation in Polyether Polyurethane: A Small-Angle X-Ray Scattering Study. J. Appl. Crystallogr..

[36] Raftopoulos K.N., Klonos P.A., Tworzydło P., Ozimek J., Hebda E., Kyritsis A., Pielichowski K. (2023). Effects of Segment Length and Crosslinking via POSS on the Calorimetric and Dynamic Glass Transition of Polyurethanes with Aliphatic Hard Segments. Int. J. Mol. Sci..

[37] Raftopoulos K.N., Janowski B., Apekis L., Pielichowski K., Pissis P. (2011). Molecular Mobility and Crystallinity in Polytetramethylene Ether Glycol in the Bulk and as Soft Component in Polyurethanes. Eur. Polym. J..

[38] Wang W., Jin Y., Su Z. (2009). Spectroscopic Study on Water Diffusion in Poly(Ester Urethane) Block Copolymer Matrix. J. Phys. Chem. B.

[39] Xiao Y., Bao L., Fu X., Wu B., Kong W., Zhou C., Lei J. (2018). Effect of Phase Separation on Water Resistance of Green Waterborne Polyurethanes: Unexpected Stronger Impact Compared to Hydrophilic Segments. Adv. Polym. Technol..

[40] Yu Q., Pan P., Du Z., Du X., Wang H., Cheng X. (2019). The Study of Cationic Waterborne Polyurethanes Modified by Two Different Forms of Polydimethylsiloxane. RSC Adv..

[41] Kanapitsas A., Pissis P., Ribelles J.L.G., Pradas M.M., Privalko E.G., Privalko V.P. (1999). Molecular Mobility and Hydration Properties of Segmented Polyurethanes with Varying Structure of Soft- and Hard-Chain Segments. J. Appl. Polym. Sci..

[42] Wang Y., Song J., Tian Q., Song N., Liang S., Tian C., Qiang X., Lei Y., Chen K., Almásy L. (2023). Understanding Water Absorption Effect on Molecular Dynamics, Microstructures and Relaxation Behavior of Segmented Polyurethane Elastomers. Polym. Degrad. Stab..

[43] Li J., Ma L., Chen G., Zhou Z., Li Q. (2015). A High Water-Content and High Elastic Dual-Responsive Polyurethane Hydrogel for Drug Delivery. J. Mater. Chem. B.

[44] Dias B., Carvalho J., Mendes J.P., Almeida J.M.M.M., Coelho L.C.C. (2022). Analysis of the Relative Humidity Response of Hydrophilic Polymers for Optical Fiber Sensing. Polymers.

[45] Bakkali-Hassani C., Berne D., Ladmiral V., Caillol S. (2022). Transcarbamoylation in Polyurethanes: Underestimated Exchange Reactions?. Macromolecules.

[46] Farshbaf M.A., Aghjeh M.K.R. (2026). Decoding Exchange Mechanisms in High-Performance Polyurethane Vitrimers: Rheological Deconvolution of Multi-Pathway Dynamics. Macromolecules.

[47] Kong Q., Tan Y., Zhang K., Zhang H., Zhu T., Wang X. (2026). Dynamic Decrosslinking Enables Self-Healing, Reprocessability, and Upcycling in Polyurethane Networks. Nat. Commun..

[48] Lee G.R., Lee E.J., Shin H.S., Kim J., Kim I., Hong S.C. (2024). Preparation of Non-Isocyanate Polyurethanes from Mixed Cyclic-Carbonated Compounds: Soybean Oil and CO_2_-Based Poly(Ether Carbonate). Polymers.

[49] Centeno-Pedrazo A., Perez-Arce J., Freixa Z., Ortiz P., Garcia-Suarez E.J. (2024). Non-Isocyanate Polyurethanes Derived from Carbonated Soybean Oil: Synthesis, Characterization and Comparison with Traditional Vegetable Oil-Based Polyurethanes. Prog. Org. Coat..

[50] Scheelje F.C.M., Meier M.A.R. (2023). Non-Isocyanate Polyurethanes Synthesized from Terpenes Using Thiourea Organocatalysis and Thiol-Ene-Chemistry. Commun. Chem..

[51] Ghorai A., Chung H. (2025). Biomass Lignin- and CO2-Based Non-Isocyanate Polyhydroxyurethanes. ACS Sustain. Chem. Eng..

[52] Shi N., Ni Y., Li Z., Chen K., Zhou D., Zhang M., Huang W., Wang Y., Chen M., Guo K. (2024). Fully Biobased Unsymmetric Bisphenols from Condensation of Lignin-Derived Monophenols for Non-Isocyanate Polyurethane Synthesis. Fuel.

[53] Díez-Poza C., Sessini V., Cabrera D.J., Mosquera M.E.G., Whiteoak C.J. (2024). Non-Isocyanate Polyurethanes (NIPUs) Incorporating Isomers of Abundant Sugar-Derived 1,4:3,6-Dianhydrohexitols. ACS Appl. Polym. Mater..

[54] Hebda E., Wróbel K., Cieślik A., Szołdrowska K., Ozimek J., Zając P., Raftopoulos K.N., Pielichowski K. (2025). Structurally Ordered NIPUs via Catalyst-Free Synthesis with Hard Segments Based on Erythritol and a Long-Chain Diamine. Molecules.

[55] Parcheta-Szwindowska P., Rohde K., Datta J. (2022). Bio-Derived Polyurethanes Obtained by Non-Isocyanate Route Using Polyol-Based Bis(Cyclic Carbonate)s—Studies on Thermal Decomposition Behavior. J. Therm. Anal. Calorim..

[56] Leitsch E.K., Beniah G., Liu K., Lan T., Heath W.H., Scheidt K.A., Torkelson J.M. (2016). Nonisocyanate Thermoplastic Polyhydroxyurethane Elastomers via Cyclic Carbonate Aminolysis: Critical Role of Hydroxyl Groups in Controlling Nanophase Separation. ACS Macro Lett..

[57] Nanclares J., Petrovic Z.S., Javni I., Ionescu M., Jaramillo F. (2015). Segmented Polyurethane Elastomers by Nonisocyanate Route. J. Appl. Polym. Sci..

[58] Leitsch E.K., Heath W.H., Torkelson J.M. (2016). Polyurethane/Polyhydroxyurethane Hybrid Polymers and Their Applications as Adhesive Bonding Agents. Int. J. Adhes. Adhes..

[59] Hu S., Chen X., Torkelson J.M. (2022). Isocyanate-Free, Thermoplastic Polyhydroxyurethane Elastomers Designed for Cold Temperatures: Influence of PDMS Soft-Segment Chain Length and Hard-Segment Content. Polymer.

[60] Wijeratne P.M., Ocando C., Grignard B., Berglund L.A., Raquez J.-M., Zhou Q. (2025). Synthesis, Thermal and Mechanical Properties of Nonisocyanate Thermoplastic Polyhydroxyurethane Nanocomposites with Cellulose Nanocrystals and Chitin Nanocrystals. Biomacromolecules.

[61] Łukaszewska I., Lalik S., Bukowczan A., Marzec M., Pielichowski K., Raftopoulos K.N. (2023). Tailoring the Physical Properties of Non-Isocyanate Polyurethanes by Introducing Secondary Amino Groups along Their Main Chain. J. Mol. Liq..

[62] Beniah G., Liu K., Heath W.H., Miller M.D., Scheidt K.A., Torkelson J.M. (2016). Novel Thermoplastic Polyhydroxyurethane Elastomers as Effective Damping Materials over Broad Temperature Ranges. Eur. Polym. J..

[63] Beniah G., Heath W.H., Jeon J., Torkelson J.M. (2017). Tuning the Properties of Segmented Polyhydroxyurethanes via Chain Extender Structure. J. Appl. Polym. Sci..

[64] Beniah G., Heath W.H., Torkelson J.M. (2017). Functionalization of Hydroxyl Groups in Segmented Polyhydroxyurethane Eliminates Nanophase Separation. J. Polym. Sci. Part A Polym. Chem..

[65] Chen X., Li L., Wei T., Torkelson J.M. (2019). Reprocessable Polymer Networks Designed with Hydroxyurethane Dynamic Cross-links: Effect of Backbone Structure on Network Morphology, Phase Segregation, and Property Recovery. Macromol. Chem. Phys..

[66] Chen X., Li L., Torkelson J.M. (2019). Recyclable Polymer Networks Containing Hydroxyurethane Dynamic Cross-Links: Tuning Morphology, Cross-Link Density, and Associated Properties with Chain Extenders. Polymer.

[67] Schimpf V., Max J.B., Stolz B., Heck B., Mülhaupt R. (2019). Semicrystalline Non-Isocyanate Polyhydroxyurethanes as Thermoplastics and Thermoplastic Elastomers and Their Use in 3D Printing by Fused Filament Fabrication. Macromolecules.

[68] Schimpf V., Heck B., Reiter G., Mülhaupt R. (2017). Triple-Shape Memory Materials via Thermoresponsive Behavior of Nanocrystalline Non-Isocyanate Polyhydroxyurethanes. Macromolecules.

[69] Li L., Zhao B., Hang G., Gao Y., Hu J., Zhang T., Zheng S. (2023). Polyhydroxyurethane and Poly(Ethylene Oxide) Multiblock Copolymer Networks: Crosslinking with Polysilsesquioxane, Reprocessing and Solid Polyelectrolyte Properties. Polymers.

[70] Chen Y., Torkelson J.M. (2024). Thermoplastic Segmented Polyhydroxyurethane: Biobased Segments Can Lead to Broad-Temperature-Range Damping Character in the Absence of Inter-Segment Hydrogen Bonding. Eur. Polym. J..

[71] Zhang K., Nelson A., Talley S., Chen M., Margaretta E., Hudson A.G., Moore R.B., Long T. (2016). Non-Isocyanate Poly(Amide-Hydroxyurethane)s from Sustainable Resources. Green Chem..

[72] Rahimzadeh R., Flanders M., Manas-Zloczower I. (2025). Optimization of the Annealing Procedure for Thermoplastic Polyurethane Systems Using in Situ Dynamic Mechanical Analysis. Phys. Fluids.

[73] Gower L.A., Lyman D.J. (1995). Phase Separation of Copolyurethanes: A Study of Annealing and Slow Solvent Evaporation Methods by FTIR. J. Polym. Sci. Part A Polym. Chem..

[74] Garrett J.T., Lin J.S., Runt J. (2002). Influence of Preparation Conditions on Microdomain Formation in Poly(Urethane Urea) Block Copolymers. Macromolecules.

[75] Hu S., Chen X., Torkelson J.M. (2019). Biobased Reprocessable Polyhydroxyurethane Networks: Full Recovery of Crosslink Density with Three Concurrent Dynamic Chemistries. ACS Sustain. Chem. Eng..

[76] Chen X., Li L., Jin K., Torkelson J.M. (2017). Reprocessable Polyhydroxyurethane Networks Exhibiting Full Property Recovery and Concurrent Associative and Dissociative Dynamic Chemistry via Transcarbamoylation and Reversible Cyclic Carbonate Aminolysis. Polym. Chem..

[77] Malewska E., Kurańska M. (2025). Biopolyolysis—A New Biopathway for Recycling Waste Polyurethane Foams. J. Environ. Chem. Eng..

[78] Nees M., Adeel M., Pazdur L., Porters M., Velde C.M.L.V., Billen P. (2024). Polyurethane Waste Recycling: Thermolysis of the Carbamate Fraction. ACS Omega.

[79] Nees M., Porters M., Adeel M., Pazdur L., Velde C.M.L.V., Billen P. (2026). Beyond the Polyol: Valorization of the Derivatized Isocyanate Fraction after Chemical Recycling of Polyurethane. RSC Sustain..

[80] Seychal G., Ocando C., Bonnaud L., De Winter J., Grignard B., Detrembleur C., Sardon H., Aramburu N., Raquez J.M. (2023). Emerging Polyhydroxyurethanes as Sustainable Thermosets: A Structure-Property Relationship. ACS Appl. Polym. Mater..

[81] Bakkali-Hassani C., Berne D., Bron P., Irusta L., Sardon H., Ladmiral V., Caillol S. (2023). Polyhydroxyurethane Covalent Adaptable Networks: Looking for Suitable Catalysts. Polym. Chem..

[82] Morinaga S., Mori Y., Tada R., Furusho Y., Kawauchi T., Endo T. (2025). Acid-Promoted Depolymerization of Poly(Hydroxyurethane)s to Five-Membered Cyclic Carbonates Toward Chemical Recycling. ChemSusChem.

[83] Purwanto N.S., Chen Y., Torkelson J.M. (2024). Biobased, Reprocessable, Self-Blown Non-Isocyanate Polyurethane Foams: Influence of Blowing Agent Structure and Functionality. Eur. Polym. J..

[84] Purwanto N.S., Chen Y., Torkelson J.M. (2023). Reprocessable, Bio-Based, Self-Blowing Non-Isocyanate Polyurethane Network Foams from Cashew Nutshell Liquid. ACS Appl. Polym. Mater..

[85] Farkhondehnia M., Maric M. (2024). Thermally Reprocessable Bio-Based Polyhydroxyurethane Vitrimers. Polymer.

[86] Bron P., Arnould N., Soares L., Wolosz D., González A., Irusta L., Sardon H., Caillol S., Ladmiral V., Bakkali-Hassani C. (2026). Catalyst-Controlled Dual Dynamic Networks in Polyhydroxyurethanes from Six-Membered Cyclic Carbonates. Macromol. Chem. Phys..

[87] Ge S., Evans C.M. (2025). Influence of Segmental Dynamics on Bond Exchange in Imine Vitrimers with Different Polymer Backbones and Cross-Linkers. Macromolecules.

[88] Lin T.W., Mei B., Dutta S., Schweizer K.S., Sing C.E. (2025). Molecular Dynamics Simulation and Theoretical Analysis of Structural Relaxation, Bond Exchange Dynamics, and Glass Transition in Vitrimers. Macromolecules.

[89] Caliari M., Vidal F., Mantione D., Seychal G., Campoy-Quiles M., Irusta L., Fernandez M., de Pariza X.L., Habets T., Aramburu N. (2025). Fully Recyclable Pluripotent Networks for 3D Printing Enabled by Dissociative Dynamic Bonds. Adv. Mater..

[90] Popov I., Cheng S., Sokolov A.P., Matyjaszewski K., Gnanou Y., Hadjichristidis N., Muthukumar M. (2022). Broadband Dielectric Spectroscopy and Its Application in Polymeric Materials. Macromolecular Engineering: From Precise Synthesis to Macroscopic Materials and Applications.

[91] Kruteva M. (2021). Dynamics Studied by Quasielastic Neutron Scattering (QENS). Adsorption.

[92] Gvaramia M., Gutfreund P., Falus P., Faraone A., Nagao M., Wolff M. (2023). Neutron Spin Echo Spectroscopy with a Moving Sample. Sci. Rep..

[93] Hemmingsen C.M., Chapman S.J., Deng C., Xiong Y., Hanley C.J., Zhang V., de la Cruz M.O., Kalow J.A. (2025). Rheological Isotope Effects for Molecular Insight in Covalent Adaptable Networks. Macromolecules.

[94] Maitra U., Chandrasekhar J. (2011). Use of Isotopes for Studying Reaction Mechanisms: 4. Distinguishing between Single Minima and Rapidly Equilibrating Structures. Resonance.

[95] Raftopoulos K.N., Łukaszewska I., Lalik S., Zając P., Bukowczan A., Hebda E., Marzec M., Pielichowski K. (2024). Structure–Glass Transition Relationships in Non-Isocyanate Polyhydroxyurethanes. Molecules.

[96] Łukaszewska I., Stachowicz K., Zając P., Pielichowski K., Raftopoulos K.N. (2024). Poly(Ethylene Oxide) Bis(Cyclic Carbonate) Based Hydrophilic Non-Isocyanate Polyhydroxyurethanes: Polymer-Water Interactions and Glass Transition Behavior. Polymer.

[97] Raftopoulos K.N., Łukaszewska I., Calduch C.B., Stachak P., Lalik S., Hebda E., Marzec M., Pielichowski K. (2022). Hydration and Glass Transition of Hybrid Non-Isocyanate Polyurethanes with POSS Inclusions. Polymer.

[98] Stachak P., Łukaszewska I., Ozimek J., Raftopoulos K.N., Bukowczan A., Hebda E., Calduch C.B., Pielichowski K. (2024). Isocyanate-Free Fabrication of Sustainable Polyurethane/POSS Hybrid Materials with Tunable Thermo-Mechanical Response. Express Polym. Lett..

[99] Sarma A.D., Zubkevich S.V., Addiego F., Schmidt D.F., Shaplov A.S., Berthé V. (2024). Synthesis of High-Tg Nonisocyanate Polyurethanes via Reactive Extrusion and Their Batch Foaming. Macromolecules.

[100] Fernández-d’Arlas B., Eceiza A. (2016). Structure-Property Relationship in High Urethane Density Polyurethanes. J. Polym. Sci. Part B Polym. Phys..

[101] Łukaszewska I., Bukowczan A., Raftopoulos K.N., Pielichowski K. (2024). Water-Polymer Interactions and Mechanisms of Water-Driven Glass Transition Decrease in Non-Isocyanate Polyhydroxyurethanes with Varying Hydration Sites. Polymer.

[102] Blahovec J., Yanniotis S. (2009). Modified Classification of Sorption Isotherms. J. Food Eng..

[103] Kwei T.K. (1984). The Effect of Hydrogen Bonding on the Glass Transition Temperatures of Polymer Mixtures. J. Polym. Sci. Polym. Lett. Ed..

[104] Łukaszewska I., Bukowczan A., Raftopoulos K.N., Pielichowski K. (2023). Examining the Water–Polymer Interactions in Non-Isocyanate Polyurethane/Polyhedral Oligomeric Silsesquioxane Hybrid Hydrogels. Polymers.

